# Orbital Hydatid Cyst, A rare cause of exophthalmos in paediatric population: A case report

**DOI:** 10.12669/pjms.41.5.11063

**Published:** 2025-05

**Authors:** Asna Tahir, Jawad Humayun, Ansa Anam

**Affiliations:** 1 Asna Tahir, Resident Ophthalmologist Department of Ophthalmology, MTI/ Khyber Teaching Hospital, Peshawar, Pakistan; 2 Jawad Humayun, FCPS Ophthalmology Department of Ophthalmology, MTI/ Khyber Teaching Hospital, Peshawar, Pakistan; 3 Ansa Anam, Resident Ophthalmologist Department of Ophthalmology, MTI/ Khyber Teaching Hospital, Peshawar, Pakistan

**Keywords:** Orbital hydatid cyst, Proptosis, Dystopia, Relative Afferent Pupillary Defect, Orbitotomy

## Abstract

**Background:**

Hydatid cyst is a parasitic infection caused by a tapeworm Echinococcus granulosus, mainly involving the liver and lungs with orbital involvement being very rare but when involved can result in unilateral proptosis leading to vision loss in chronic cases. Here, we present a rare case of unilateral orbital hydatid cyst in young male patient of pediatric age group which was successfully treated.

**Case report:**

A four years old male patient presented with history of trauma and unilateral painless temporal proptosis with dystopia of right eye for two months. On ocular examination, his visual acuity in the right eye was 6/60 and in the left eye it was 6/6. In his right eye there was a positive Relative Afferent Pupillary Defect. On fundus examination, the optic disc was swollen in right eye. The dystopia was 10 mm each, laterally and inferiorly, there was also resistance to retropulsion. In the right eye extraocular movements were restricted in all gazes, a soft tender mass was palpable superior-medially and mild lagophthalmos was also present. Magnetic resonance imaging findings led to the diagnosis of orbital hydatid cyst being the cause of proptosis. Surgical removal of the cyst through superior orbitotomy was performed and its contents were aspirated under general anesthesia. The diagnosis of orbital hydatid cyst was confirmed by histopathological reports of the cyst walls and the aspirated fluid.

**Conclusion:**

Orbital hydatid cyst is a very rare occurrence and should be considered a differential diagnosis of proptosis in pediatric population. Surgical removal of the cyst is the main treatment option followed by oral Albendazole for three months.

## INTRODUCTION

Hydatid cyst, a parasitic infection primarily involving liver and lungs is caused by Echinococcus granulosus. It is endemic in Southern Europe, Turkey, Australia, New Zealand, South America, Africa, India and Middle East.^1^ The definitive hosts for the Echinococcus granulosus are the dogs whereas cattle such as sheep are the intermediate hosts.^2^ The accidental intermediate hosts affected by the tapeworm are humans, who after ingesting the parasitic viable eggs become intermediate hosts. In the human body, during its life cycle, Echinococcus granulosus affects the liver and lungs commonly. Involvement of liver is 55-70% and that of lungs is 18-35%.^3^ The rarest of presentations of hydatid cyst is its occurrence in the orbit which accounts for about 1% of the total cases of hydatid disease.^4^ Due to the orbital involvement patients may present with complaints of proptosis, decreased vision and ocular pain.^5^

In case of orbital involvement, it is most commonly located in intraconal compartment occupying the superomedial or superolateral part of the orbit, although less common but it can also be seen in the extraconal compartment. Moreover, in rare instances, it can occupy the orbital floor thus pushing the eyeball in the superior direction and forward.^6^ A large cyst occupying space in the orbit may lead to optic nerve compression thus causing restricted extraocular movements. Magnetic Resonant Imaging (MRI) can help in diagnosis but only histopathology of the cyst can give a definitive diagnosis. The main treatment of orbital hydatid cyst is surgical excision, followed by a course of oral Albendazole (10mg/kg) for 12 weeks.^7^ Here, we present a rare case of unilateral orbital hydatid cyst in young male patient of pediatric age group who was successfully treated.

## CASE PRESENTATION

**Fig.1:**
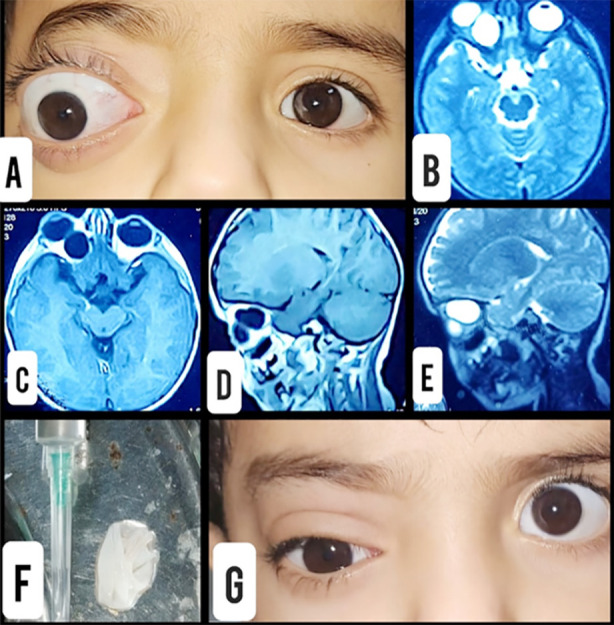
**A.** Right proptosis at presentation. **B.** T2 Axial MRI Scan. **C.** T1 Axial MRI Scan. **D.** T1 Sagittal MRI Scan. **E.** T2 Sagittal MRI Scan. **F.** Excised cyst wall with aspirated fluid. **G.** First Post-Operative Day.

A four year old male patient visited The Ophthalmology department. He was examined in the out-patient department and was admitted for proptosis work up in ophthalmology department. Physical and ocular examinations were carried out. The patient was vitally stable and well oriented in person, place and time. He had history of trauma two months ago. He presented with Proptosis and Inferior dystopia to OPD of Eye Department. (Fig.1A) outpatient department with the chief complaint of unilateral painless proptosis along with dystopia of the right eye since past two months. His visual acuity in the right eye was 6/60 and in the left eye, it was 6/6. Pupils were round in shape bilaterally. RAPD (Relative Afferent Pupillary Defect) was positive in the right eye. The dystopia was measured to be 10 mm laterally and 10 mm inferiorly. Hertel Exophthalmometer was used to measure proptosis. The Exophthalmometer measurement for the right eye was 27 mm whereas it was 19 mm for the left eye. There was no increase in exophthalmos with crying, straining and Valsalva maneuver. There was no bruit and the intraocular pressure (IOP) was normal bilaterally.

A palpable mass was present in the right orbit superior-medially. Retropulsion test was performed on both eyes, resistance was felt in the right eye due to space occupying mass. Extra-ocular movements of the right eye were restricted in all gazes whereas they were full in the left eye. Mild lagophthalmos was also present in the right eye. On slit lamp examination, the cornea and conjunctiva were clear and anterior chamber was deep and quiet. A series of tests were run and all the relevant specialties were consulted. All the serological tests including the CBC (complete blood count), (RFTs) renal function tests, LFTs (liver function tests, ESR (erythrocyte sedimentation rate) were well within normal ranges. Radiological investigations were conducted.

On ultrasound abdomen three large unilocular cystic lesions measuring 5.1 cm, 4.7 cm and 5.2 cm in diameter in the right lobe of liver were seen, these cysts showed thick double wall and no internal septation, solid component or calcification, all these findings were suggestive of hydatid cysts. Rest of the ultrasound was normal. A brightness scan (B scan) of the right eye was done which showed clear fluid containing cystic mass superior-medially to the right eyeball. An MRI of brain and orbits was done in which a benign intraocular mass lesion measuring approximately about 19 x 17 x 16 mm, most likely suggestive of orbital hydatid cyst was mentioned (Fig.1B, 1C, 1D, 1E). Computerized tomography scan (CT scan) of brain showed defect in the superior orbital roof with associated extraconal fluid density collection along the superior-medial aspect of right orbit. Rest of the investigations were unremarkable.

The patient was operated for the removal of cyst under general anesthesia. Cyst was removed by performing superior orbitotomy and its contents were aspirated. (Fig.1F) The cyst walls along with the aspirated contents were sent for histopathological examination, the results of which came out to be positive for hydatid cyst. The post-operative visual acuity in the right eye of the patient improved from 6/60 to 6/6. Moreover, extra-ocular movements of the right eye became full in all gazes and the swelling of the optic disc decreased. The proptosis was completely reversed. (Fig.1G) He was started on albendazole therapy for three months.

## DISCUSSION

Echinococcus granulosus commonly known as a tapeworm is the causative organism of the orbital hydatid cyst. Of the total incidence, orbital involvement comprises less than 1%.^4^ According to the medical literature, due to the path of left carotid artery, hydatid cyst affects the left orbit mainly.^8^ However, in this case report, the right eye of the four year old male patient was effected. Unilateral proptosis which is progressive in nature is the most frequent presentation with the proptosis usually being quite significant. Due to limited space of bony orbit, when the cyst enlarges the intra orbital pressure also increases gradually resulting in the compression of the optic nerve which the causes swelling of the optic disc, RAPD and a decrease in vision. In this case report, the patient had right swollen optic disc and a positive RAPD in his right eye, whereas the left eye was normal. Imaging studies including MRI of the orbit, Ct-scan and B-scan help in presumptive diagnosis.

MRI orbit demonstrates a homogeneous lesion, which is hypo-intense on T1 weighted images (Fig.1C, 1D) and hyper-intense on T2 weighted images (Fig.1B, 1E). Regarding location of the hydatid cyst in the orbit, it was extraconal in our patient which makes it a rare instance since in literature it is mentioned that most of the cysts are intraconal.^9^ Medical literature describes various approaches and surgical methods. Orbital surgeons perform orbitotomy whereas Neurosurgeons perform craniotomy and orbitotomy.^10^ Lack of long-term follow-up was the major limitation in this case report, since our patient was from Afghanistan and could not come regularly for follow-ups.

## CONCLUSION

Among the differential diagnosis of unilateral proptosis, orbital hydatid cyst, although rare, should also be included. Radiological studies such as MRI, CT-scan and B-scan can give presumptive diagnosis of hydatid cyst but the definitive diagnosis is through histopathological examination of the cyst. Treatment of choice is surgical excision with extraordinarily good postoperative results, provided surgical intervention is done timely and excision of cyst is performed earlier, followed by oral Albendazole for 12 weeks.

### Author’s Contribution:

**ATahir:** Conceived, designed, drafted & edited manuscript, is responsible for integrity of research.

**ATahir, JH & AAnam:** Revised it critically for important intellectual content.

**JH:** Did review and final approval of manuscript.
